# Eye Movements But Not Vision Drive the Development of Palisade Endings

**DOI:** 10.1167/iovs.63.11.15

**Published:** 2022-10-18

**Authors:** Genova Carrero-Rojas, Paula M. Calvo, Thomas Lischka, Johannes Streicher, Rosa R. de la Cruz, Angel M. Pastor, Roland Blumer

**Affiliations:** 1 Center of Anatomy and Cell Biology, Medical University Vienna, Vienna, Austria; 2 Departament of Physiology, Faculty of Biology, University of Seville, Seville, Spain; 3 Department of Ophthalmology, Carl von Ossietzky University of Oldenburg, Oldenburg, Germany; 4 Department of Anatomy and Biomechanics, Division of Anatomy and Developmental Biology, Karl Landsteiner University of Health Science, Krems an der Donau, Austria

**Keywords:** eye muscles, proprioception, palisade endings, botulinum neurotoxin

## Abstract

**Purpose:**

To test whether visual experience and/or eye movements drive the postnatal development of palisade endings in extraocular muscles.

**Methods:**

In three newborn cats, the right eye was covered until 30 days from postnatal (P) day 7 (before opening their eyes), and in three cats both eyes were covered until 45 days, also from P7. To block eye movements, another seven cats received a retrobulbar injection of botulinum neurotoxin A (BoNT-A) into the left orbit at birth and survived for 45 days (three cats) and 95 days (four cats). The distal third of the rectus muscles containing the palisade endings was used for whole-mount preparation and triple-fluorescence labeling with anti-neurofilament along with (1) anti-synaptophysin and phalloidin or (2) anti-growth associated protein 43 (GAP43) and phalloidin. Immunolabeled specimens were analyzed in the confocal laser scanning microscope.

**Results:**

After unilateral and bilateral dark rearing, palisade endings were qualitatively and quantitatively equal to those from age-matched controls. After BoNT-A induced eye immobilization for 45 or 95 days, palisade endings were absent in the superior rectus and lateral rectus muscles and only present in the inferior rectus and medial rectus muscle. These BoNT-A–treated palisade endings were rudimentary and reduced in number, and the expression of the neuronal developmental protein GAP43 was significantly reduced.

**Conclusions:**

This study demonstrates that eye immobilization, but not visual deprivation, affects palisade ending development. Palisade endings develop in the first month of life, and the present findings indicate that, during this time window, palisade endings are prone to oculomotor perturbations.

The eyes are the most mobile organs of the body, and for visually guided behavior one must know in which direction the eyes are pointing. It is supposed that specialized sense organs (proprioceptors) transmit signals about the length and tension of extraocular muscles (EOMs) to the brain, and with that information the brain computes the eye's position. Although there is evidence that afferent signals from EOMs reach the brain,^1^^,^^2^ the classical proprioceptor pair (muscle spindles and Golgi tendon organs) is absent in the EOMs of most mammals with the exception of even-toed ungulates, where they occur at very high numbers (e.g., pig, cow, sheep).^3^ In human EOMs, muscle spindles, albeit with a simplified morphology, are present but not Golgi tendon organs.^4^^,^^5^ Because of the discrepancies between findings in the effector organ (EOM) and the brain, proprioception is still an unresolved issue in the field of visual science.

Candidates for EOM proprioception are palisade endings, which are virtually present in all mammalian orders analyzed so far, except rodents.^6^^–^^12^ Palisade endings are peripheral specializations of axons with exuberant nerve terminals that surround individual muscle fiber tips at the muscle–tendon junction.^6^ Structurally, palisade endings have similarities to sensory Golgi tendon organs, but molecular and connectional features indicate that they are more compatible with motor structures. Specifically, palisade endings are cholinergic,^13^^–^^15^ express key proteins involved in neurotransmitter (acetylcholine) release,^16^ and originate from the EOM motor nuclei, most likely from neurons whose axons establish multiple motor endings (en grappe motor terminals) along the so-called multiply innervated muscle fibers (MIFs).^17^^,^^18^ Because sensory and motor features coexist in palisade endings, their function is still elusive.

We have shown in a frontal-eyed species (cat) that palisade endings develop after birth in a muscle-specific heterochronic sequence to reach a different final density per individual muscle.^19^ That is, palisade endings mature earlier in the medial rectus muscle (they exhibit adult characteristics by 45 days after birth) and later in the other rectus muscles; additionally, many more palisade endings are present in the medial rectus muscle compared with the other rectus muscles.^19^ Cats develop a complex visuomotor behavior in the first month of life, and this requires visual experience and visually guided eye movements.^20^^,^^21^ Because the development of palisade endings and visuomotor coordination is almost parallel, we hypothesized that visual experience that occurs after eye opening around day eight after birth and/or eye movements are important for palisade ending maturation.

To test both hypotheses and to sort out which of them is more relevant, two experiments (visual deprivation and eye immobilization) were independently performed in different animals. In the visual deprivation experiment, the eyes of kittens were unilaterally or bilaterally covered with a mask just before eye opening, which was around 8 days after birth. In the eye immobilization experiment, newborn cats received unilateral retrobulbar botulinum neurotoxin A (BoNT-A) injection into the space behind the bulbus oculi. BoNT-A is a neurotoxin that, by blocking neurotransmission, leads to muscle paralysis.^22^^–^^26^ Our results show that eye immobilization, but not visual deprivation, significantly delays palisade ending maturation. Additionally, following eye immobilization, the number of palisade endings was reduced.

## Materials and Methods

### Animals

Fourteen cats (13 experimental animals and one age-matched control) were used in this study. Additionally, a cat from a previous study of our research group was used.^19^ Handling procedures for experiments followed the guidelines of the National Institutes of Health (http:/oacu.od.nih.gov) and specific recommendations for maintenance of higher mammals during neuroscience experiments (NIH publication #94-3207, 1994), and they were in accordance with Spanish legislation for the use and care of laboratory animals (R.D. 53/2013, BOE 34/11370-421, 2013). The study was approved by the local ethics committee. All efforts were made to keep the number of animals used low in the present study.

### Experimental Design

#### Unilateral/Bilateral Visual Deprivation

**Figure 1.**
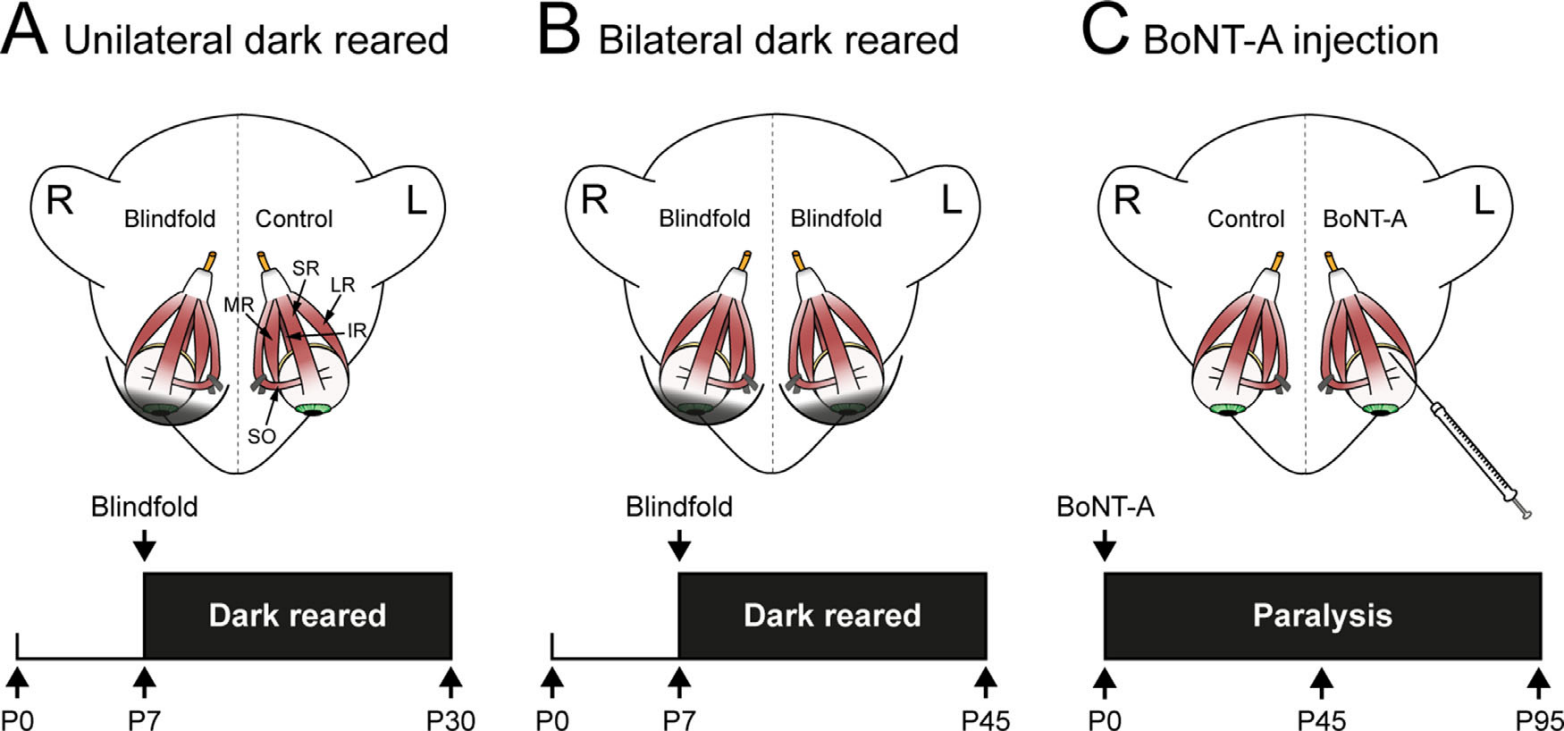
The experimental design of the study. (**A**) Schematic drawing showing the unilateral visual deprivation. In this situation, only one eye (right side) was covered with a blindfold at P7, and the animals were sacrificed at P30. (**B**) Diagram illustrating bilateral visual deprivation. In this case, both eyes were covered with a blindfold at P7, just before cats open their eyes (P8). These animals were sacrificed at P45. (**C**) Retrobulbar BoNT-A injection into the left orbit at birth (P0). The survival time was 45 days for the first group and 95 days for the second group. SR, superior rectus; LR, lateral rectus; IR, inferior rectus; SO, superior oblique; MR, medial rectus muscle.

Six cats were used for the visual deprivation experiments. Unilateral and bilateral visual deprivation was performed in each of three animals, respectively (Figs. 1A, 1B). In animals with unilateral (right eye) visual deprivation, the contralateral eye served as control. In the bilateral visual deprivation experiment, one age-matched animal of the same litter grew up with light experience and served as control. Visual deprivation started at postnatal (P) day 7, just before the cats opened their eyes (around P8). The eyes were covered with a double linen pad and a custom-made mask with surgical tape that secured the covered eye. The mask was removed every three days in a dim environment in a procedure that lasted no longer than 2 minutes to inspect and, if needed, clean the eye and the face of each animal. This procedure followed the recommendations of Kalina Burnat (Nencki Institute of Experimental Biology, Poland). Unilaterally visually deprived animals were sacrificed at P30 (Fig. 1A), and bilaterally visually deprived animals were sacrificed at P45 (Fig. 1B).

#### Eye Immobilization

Seven animals were used for eye immobilization. At birth, cats received a single unilateral injection of BoNT-A into the space behind the left bulbus oculi (retrobulbar space). (BoNT-A was kindly provided by Michael-Robert Popoff from the Pasteur Institute with the usage authorization of the Agence Nationale de Sécurité du Médicament et des Produits de Santé of France.) Five animals received the retrobulbar injection superior to the globe, and two animals received the retrobulbar injection inferior to the globe with no differences in the results. The right orbit received no BoNT-A injection and served as control. Aliquots were stored at −80°C and, on the day of use, were freshly diluted in phosphate buffer (0.1 M, pH 6.3) to a final concentration of 54 pg/µL. A total of 50 µL was retrobulbarly injected in the left orbit (Fig. 1C) using an Omnican 50 insulin syringe with a 30-gauge integrated needle (B. Braun Melsungen AG, Melsungen, Germany). This volume represents a 2.7-ng of BoNT-A dose per animal, which had an average weight of approximately 100 g at the day of birth (27 ng/kg). Three animals had a survival time of 45 days and four animals of 95 days.

### Tissue Preparation and Processing

Cats were deeply anesthetized with a mixture of ketamine and xilacine at 20 mg/kg and 1 mg/kg intramuscularly, respectively. Then, a terminal dose of sodium pentobarbital (100 mg/kg intraperitoneally) was administered for intracardial perfusion with physiological saline followed by 4% paraformaldehyde in 0.1-M phosphate buffer at pH 7.4. The eyeballs, including the EOMs, were removed, and the rectus muscles (superior rectus, inferior rectus, lateral rectus, and medial rectus muscle) were dissected free. Muscles were cut transversally into two pieces: one piece consisting of the distal part of the muscle with the attached tendon and the other piece consisting of the muscle belly. The distal muscle–tendon junctions (site of the palisade endings) were stored at 4°C in PBS (0.1 M, pH 7.4) containing 0.05% sodium azide to avoid bacterial and fungal contamination. They underwent whole-mount preparation. The muscle bellies were used for control experiments and were divided into two groups. One group of muscle bellies was cryoprotected in graded concentrations of sucrose (10%, 25%, and 40%) in PBS, frozen, and stored at −80°C. A second group of muscle bellies was immersion fixed in modified Karnovsky solution containing 2% paraformaldehyde and 2.5% glutaraldehyde in 0.1-M PBS at pH 7.4 for 24 hours at 4°C. Following rinsing in PBS, tissue was postfixed in 1% osmium tetroxide in PBS for 12 hours at 4°C, dehydrated in graded dilutions of ethanol, and embedded in Epon. All distal myotendons and the first set of muscle bellies underwent multicolor immunofluorescence. The Epon-embedded second set of muscle bellies underwent histological and transmission electron microscopic (TEM) analyses.

### Multicolor Immunofluorescence

#### Antibodies and Toxins

All primary antibodies were obtained from MilliporeSigma (Burlington, MA, USA) and included chicken anti-neurofilament (RRID no. AB_11212161), mouse anti-synaptophysin (RRID no. AB_94786), goat anti-choline acetyltransferase (anti-ChAT; RRID no. AB_2079751), and rabbit anti-growth associated protein 43 (anti-GAP43, RRID number AB_109488). Chicken anti-neurofilament was used at a concentration of 1:2000, mouse anti-synaptophysin at 1:400, goat anti-ChAT at 1:100, and rabbit anti-GAP43 at 1:500. Secondary antibodies and toxins were obtained from Thermo Fisher Scientific (Waltham, MA, USA). Secondary antibodies were raised in goat or rabbit and conjugated with Alexa Fluor 568 or Alexa Fluor 488. They were used at a concentration of 1:500. Two toxins, Alexa Fluor 647-conjugated phalloidin and Alexa Fluor 488-conjugated α-bungarotoxin, were used at concentrations of 1:150 and 1:500, respectively.

#### Double Fluorescence Labeling

Muscle bellies were doubly labeled with anti-ChAT and α-bungarotoxin. Anti-ChAT visualizes cholinergic axons and α-bungarotoxin acetylcholine receptors of motor endplates. Before immunolabeling, cryosections (200-µm thickness) parallel to the muscle surface were cut. Sections were blocked for 1 hour with 10% normal rabbit serum followed by incubation with the primary antibody, goat anti-ChAT, for 48 hours at 4°C. After washing in PBS containing 0.1% Triton X-100 (PBS-T), sections were incubated with the secondary antibody along with α-bungarotoxin for 2 hours at 37°C. Sections were rinsed again and coverslipped with Dako fluorescence mounting medium (Agilent, Santa Clara, CA, USA).

#### Triple Fluorescence Labeling

Two triple-labeling experiments were performed in EOM whole-mount preparations of the distal muscle-tendon junctions: (1) labeling with anti-neurofilament and anti-synaptophysin along with phalloidin and (2) labeling with anti-neurofilament and anti-GAP43 along with phalloidin. Anti-neurofilament is a pan neuronal marker,^27^ and anti-synaptophysin is a marker for nerve terminals.^28^ Anti-GAP43 visualizes nerve fibers during development and regeneration,^29^ and phalloidin visualizes muscle fibers.

Before antibody application, the tissue was blocked for 2 hours with 10% normal goat serum. Then, the tissue was incubated for 48 hours with the primary antibodies diluted in PBS-T. Following extensive washing in PBS-T, tissue was incubated for 6 hours with the secondary antibodies along with phalloidin. Finally, the tissue was rinsed again and mounted in v/v 60% glycerin + 40% PBS. A more detailed description of immunolabeling of EOM whole-mounts is provided elsewhere.^16^ For negative controls, primary antibodies were omitted, and secondary antibodies were used alone. In all cases, the omission of the primary antibodies resulted in a complete lack of immunostaining.

### Data Analyses of Immunofluorescence

**Figure 2.**
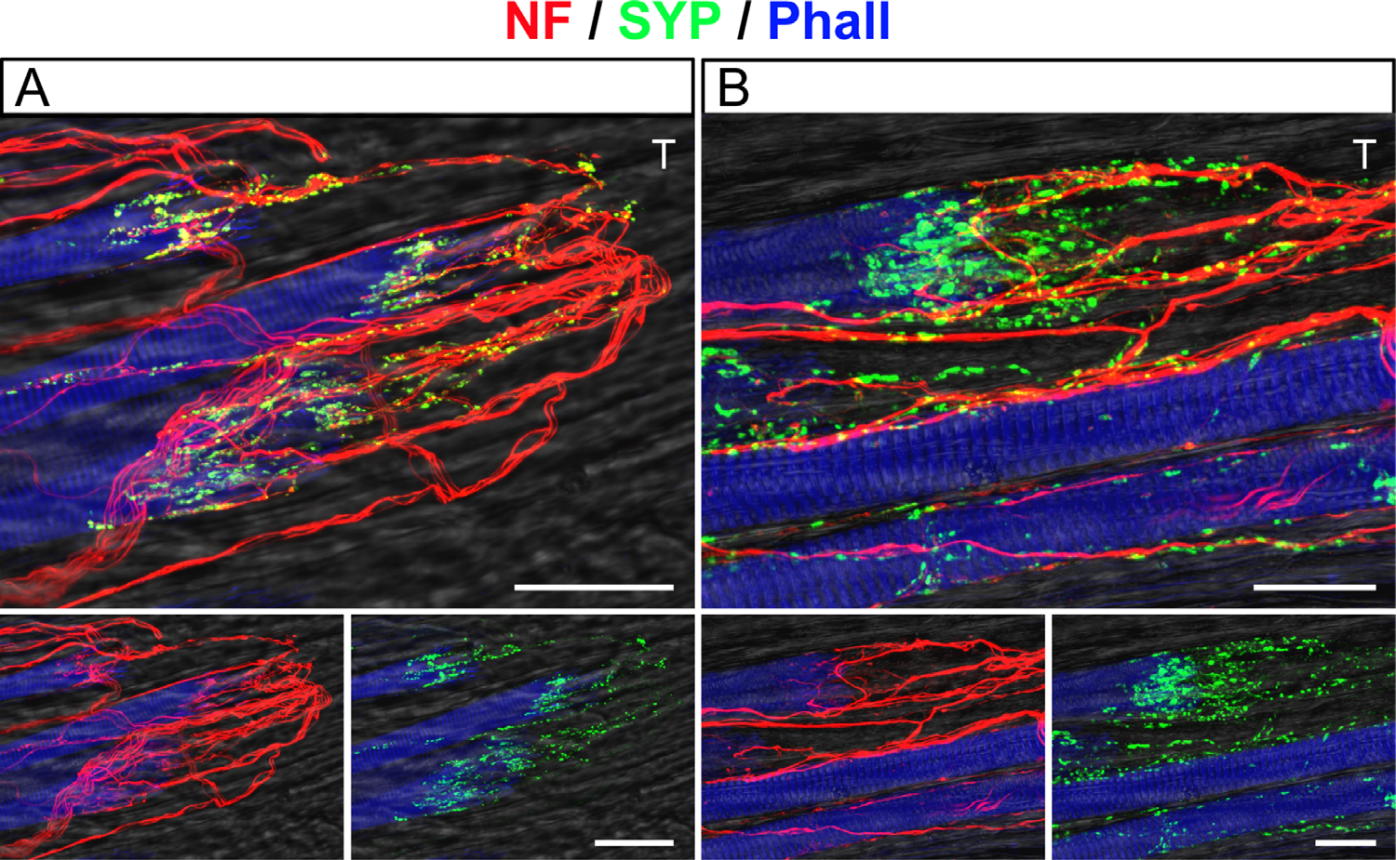
Structural features of palisade endings. (**A**, **B**) Three-dimensional projections of CLSM images showing palisade endings at lower (**A**) and higher (**B**) magnification. Palisade endings are labeled with anti-neurofilament (NF, *red*) and anti-synaptophysin (SYP, *green*). Muscle fibers are stained with phalloidin (Phall, *blue*). The tendon (T) continues to the right from the muscle fibers and is visualized in brightfield. The same staining setup is found in Figures 3, 4, 6 to 8, and 10. Palisade endings are formed by nerve fibers that come from the muscle and extend into the tendon, where they make a U-shaped turn to approach individual muscle fiber tips. By further branching, axons establish synaptophysin-positive terminal varicosities at the level of the muscle fiber tips and tendon. In the lower part of **A** and **B**, the color channels are separated to illustrate either neurofilament (in *red*) or synaptophysin (in *green*) in addition to phalloidin. *Scale bars*: 50 µm in **A** and 25 µm in **B**.

Fluorescently labeled muscle belly sections and EOM whole-mounts were analyzed with a confocal laser scanning microscope (CLSM; Olympus FV3000; Olympus Europa, Hamburg, Germany). A series of virtual CLSM sections of 1-µm thickness were cut through the structures of interest. Each section was photodocumented at a 1024 × 1024-pixel resolution, and three-dimensional (3D) projections were rendered using Image J software (National Institutes of Health, Bethesda, MA, USA). Double-colored images (see Figs. 5G, 5G’, 5H, 5H’) were generated using lasers with excitation wavelengths of 488 nm and 568 nm, and triple-colored images (Figs. 2^3^–4; see also Figs. 6^7^,^8^,^9^–10) were produced using an additional laser with an excitation wavelength of 633 nm. In some cases, brightfield images were recorded with a laser (excitation wavelength 488 nm) to correlate immunolabeling and morphological structures (Fig. 2; see also Figs. 8C, 8C’).

**Figure 3.**
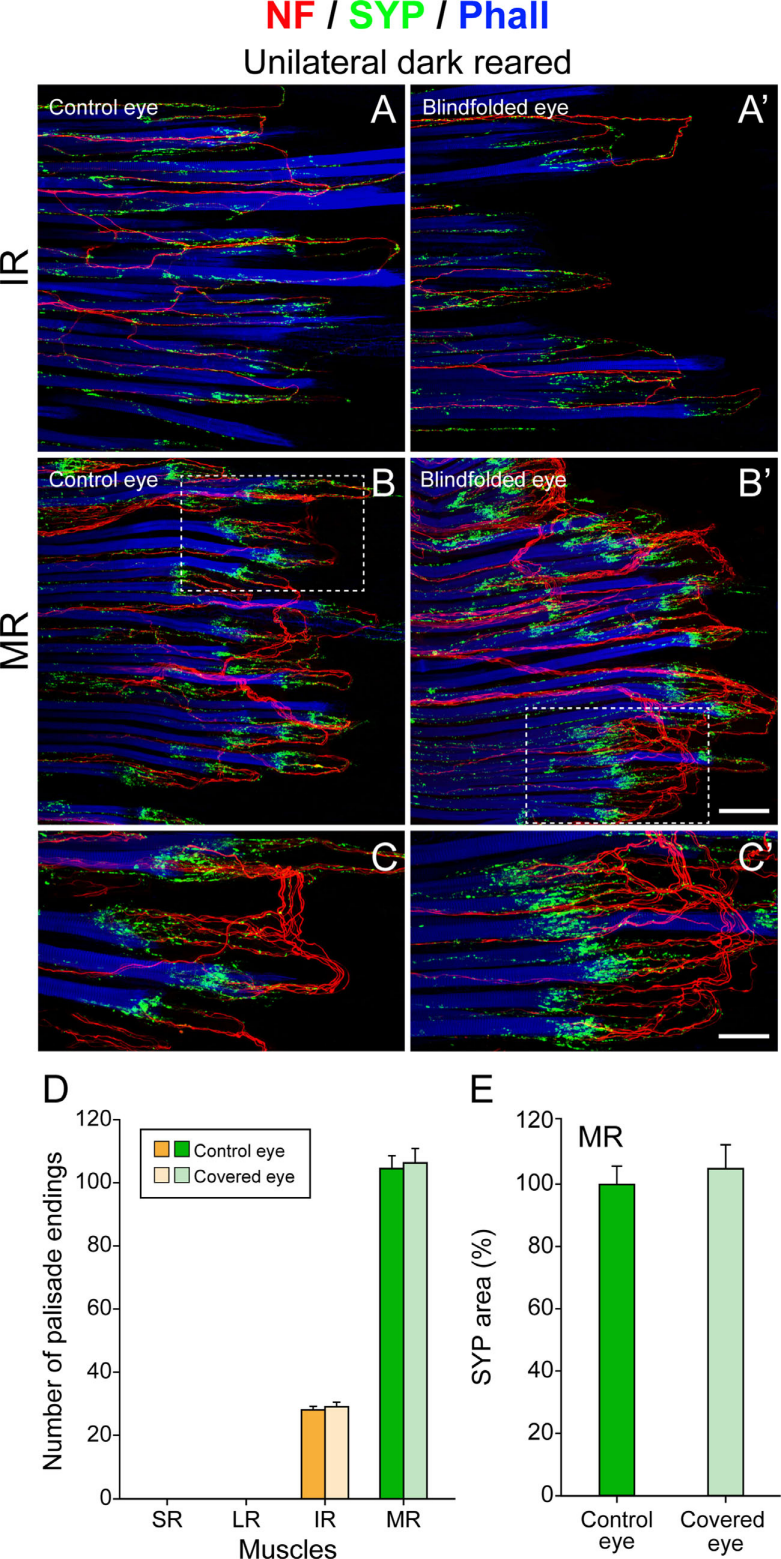
Structure and quantification of palisade endings following unilateral visual deprivation until postnatal age P30. (**A**–**C**, **A’**–**C’**) Three-dimensional projection of palisade endings in neurofilament (NF, *red*), synaptophysin (SYP, *green*), and phalloidin (Phall, *blue*) staining. The tendon is not visualized and continues to the right from the muscle fibers. Palisade endings are shown for the uncovered eye (**A**–**C**) and covered eye (**A’**–**C’**) at low magnification (**A**, **A’**, **B**, **B’**) and higher magnification (**C**, **C’**). Palisade endings in the inferior rectus (IR) muscle (**A**, **A’**) and medial rectus (MR) muscle (**B**, **B’**, **C**, **C’**) exhibit no structural differences between the uncovered and covered eye. Because palisade endings develop heterochronically, they are simpler in the IR muscle (**A**, **A’**) and more complex in the MR muscle (**B**, **B’**, **C**, **C’**), as indicated by a higher degree of axonal branching and number of synaptophysin-labeled boutons. (**D**) Bar chart showing that the number of palisade endings varies among individual rectus muscles, but there are no side differences. At the age of P30, palisade endings were absent in the lateral rectus (LR) and superior rectus (SR) muscles, and many more palisade endings are present in the MR muscle than the IR muscle. Mean ± SEM for palisades in rectus muscles of the control/covered eye: IR, 28.3 ± 0.9/29.3 ± 1.5 (*t*-test, *t*(4) = −0.588, *P* = 0.588); MR, 106.7 ± 3.8/108.7 ± 5.8 (*t*-test, *t*(4) = −0.288, *P* = 0.788). (**E**) Bar chart showing that the areas of terminal varicosities in palisade endings of the medial rectus muscle in the control/covered eye following unilateral visual deprivation show no significant differences. Mean ± SEM for control/covered eye: 100.0 ± 5.5/105.6 ± 7.4 (*t*-test, *t*(4) = −0.604, *P* = 0.579). *Scale bars*: 100 µm in **B’** (for **A**, **A’**, **B**, **B’**) and 50 µm in **C’** (for **C**, **C’**).

**Figure 4.**
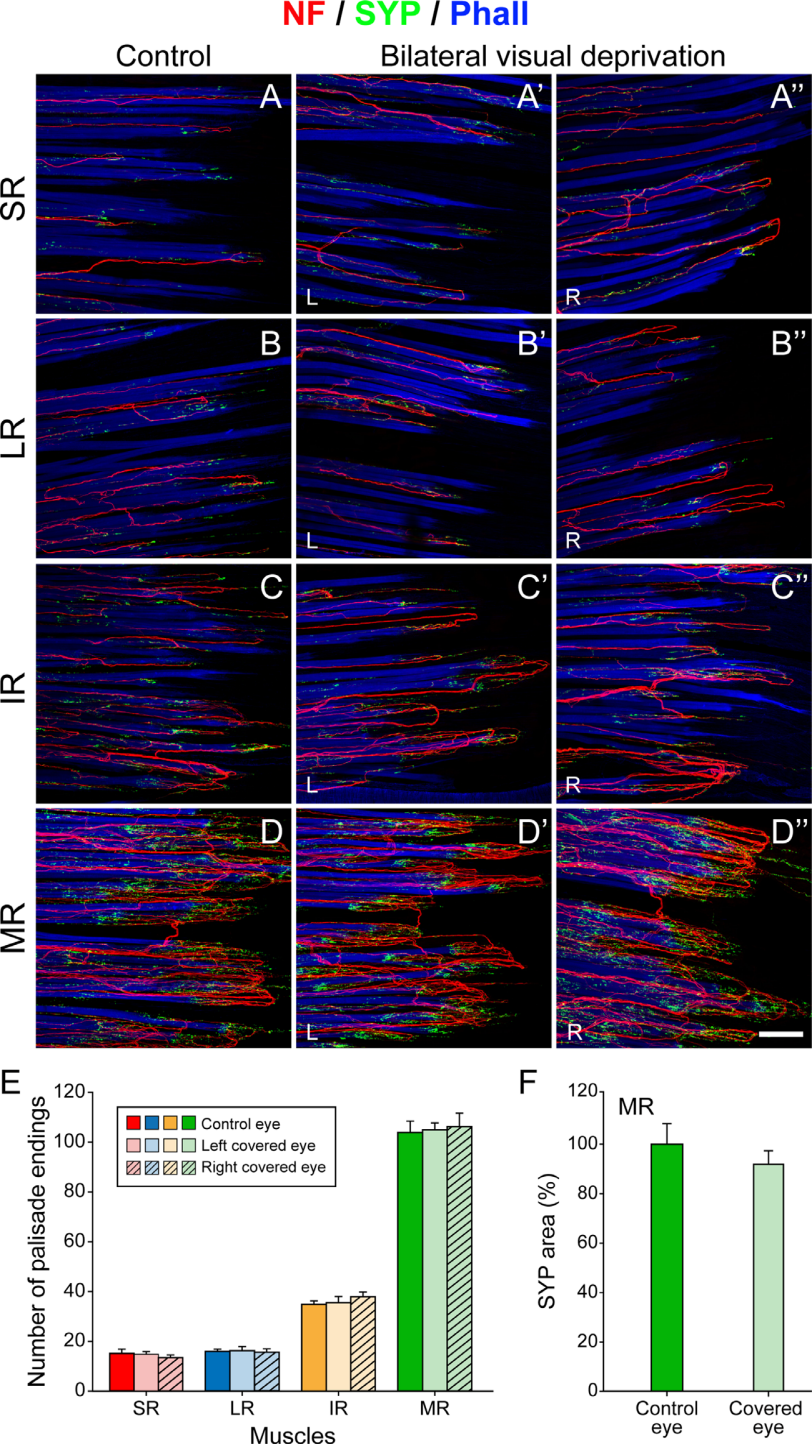
Structure and quantification of palisade endings after bilateral visual deprivation until postnatal age P45. (**A**–**D**, **A’**–**D’**, **A”**–**D”**) Three-dimensional projection of palisade endings in neurofilament (NF, *red*), synaptophysin (SYP, *green*), and phalloidin (Phall, *blue*) staining. Palisade endings are visualized in control animals (**A**–**D**) and in the left and right eyes of visually deprived animals (left side, L: **A’**–**D’**; right side, R: **A”**–**D”**). Palisade endings are formed by nerve fibers that extend into the tendon where they turn back to establish synaptophysin-positive terminal varicosities around individual muscle fiber tips. In P45 controls, palisade endings are less developed in the superior rectus (SR) muscle (**A**) and lateral rectus (LR) muscle (**B**), and they exhibit fewer axonal branches and fewer terminal varicosities, as demonstrated by low levels of synaptophysin. More developed palisade endings with a higher degree of axonal branching and higher levels of synaptophysin-positive terminal varicosities are present in the inferior rectus (IR) muscle (**C**). Almost adult-like palisade endings with complex axonal branching and high synaptophysin levels are present in the medial rectus (MR) muscle (**D**). Following visual deprivation, palisade endings in the SR muscle (**A’**, **A”**), LR muscle (**B’**, **B”**), IR muscle (**C’**, **C”**), and MR muscle (**D’**, **D”**) exhibit no differences with respect to side and are qualitatively equal to palisade endings of controls. (**E**) Bar chart showing that the number of palisade endings varies in individual rectus muscles with no significant differences between controls and visually deprived animals for each muscle. The lowest density of palisade endings is in the SR and LR muscles and the highest in the MR muscle, whereas values for the IR muscle are in between. Mean ± SEM for palisades in the rectus muscles of the control/left covered/right covered eye: SR, 15.0 ± 1.5/14.7 ± 0.9/13.3 ± 0.3 (one-way ANOVA test, *F*(2,6) = 0.724, *P* = 0.523); LR, 16.0 ± 0.6/16.3 ± 1.5/15.7 ± 1.2 (one-way ANOVA test, *F*(2,6) = 0.086, *P* = 0.919); IR, 35.0 ± 1.2/35.7 ± 2.3/38.0 ± 1.7 (one-way ANOVA test, *F*(2,6) = 0.761, *P* = 0.507); MR, 104.0 ± 4.4/105.0 ± 2.6/106.3 ± 5.2 (one-way ANOVA test, *F*(2,6) = 0.077, *P* = 0.926). (**F**) Bar chart showing that the percentage of the area of terminal varicosities in palisade endings of MR muscles in control and covered eyes shows no significant difference. Mean ± SEM: 100.0 ± 10.3 for control eye, 92.1 ± 5.0 for left covered eye (*t*-test, *t*(4) = 0.690, *P* = 0.528). *Scale bar*, 100 µm in **D”** (for all images and **D”**).

### Transmission Electron Microscopy

Muscle bellies were embedded in Epon and used for TEM. Semithin cross-sections at 1-µm thickness were cut with a Leica Ultracut UCT (Leica Camera, Wetzlar, Germany), stained with toluidine blue, and viewed under the light microscope. When structures of interest were detected at light microscopic level, ultrathin sections were cut, mounted on dioxane–formvar-coated copper grids, immersed for 10 minutes in an aqueous solution containing 2% uranyl acetate followed by 3 minutes of a solution of 0.4% lead citrate, and then examined under a Philips CM10 TEM (Philips, Amsterdam, Netherlands).

### Morphological Analyses

Morphological analyses were performed in control and experimental animals including unilateral visual deprivation, bilateral visual deprivation, and BoNT-A injection (n = 3 in unilateral/bilateral visual deprivation and n = 7 in BoNT-A experiments). The following analyses were done using z-stacks of captured confocal images: (1) quantification of palisade endings, (2) measurement of the area covered by synaptophysin signals in palisade endings, and (3) measurement of the GAP43 optical density in palisade endings.

For quantification, palisade endings were visualized with anti-neurofilament, anti-synaptophysin, and phalloidin and viewed with 20× magnification. Because each palisade ending has a cuff of synaptophysin-positive terminal varicosities at the muscle fiber tip, we counted those cuffs to determine the number of palisade endings per muscle. For determining the synaptophysin density in palisade endings, the total fluorescent area of synaptophysin across the entire image captured (1024 × 1024 pixels) with a 40× oil-immersion objective was measured (n = 3 images per experimental situation). In each image, identical thresholds were set for synaptophysin signals, and then the area covered by synaptophysin was measured using the Analyze Particles tool of Image J. The synaptophysin area was set at 100% in controls, and values in experimental animals were presented as percentage of control values. For the measurement of the GAP43 optical density, a P95 animal was used. Images of palisade endings were captured at 40× magnification with the oil-immersion objective. In each image, 50 squares of approximately 6 × 6 μm were selected in nerve fibers forming palisade endings and in the palisade endings themselves to measure the intensity of the GAP43 signal (i.e., the optical density). For background correction, five optical density values of the same area were selected from non-stained areas. Then, the background value was subtracted, and the values of the GAP43 optical density were normalized with respect to the control side of the animal. The GAP43 intensity was set at 100% on the control side and values at the BoNT-A side were presented as percentage of the control values.

### Statistical Analysis

The number of palisade endings after bilateral visual deprivation was statistically compared using the one-way ANOVA test at a level of significance of P < 0.05. The number of palisade endings after unilateral visual deprivation and BoNT-A administration, the synaptophysin area, and the GAP43 optical density between the control and experimental sides were statistically analyzed using the Student’s t-test at a level of significance of P < 0.05. For the muscle weight, the paired Student’s t-test was used for comparisons between the control and the BoNT-A sides. All values are expressed as the mean ± standard error of the mean (SEM). Statistical analyses were carried out in Sigma Plot 11 (Systat Software, San Jose, CA, USA).

## Results

### Structure of Palisade Endings

For better understanding, we first describe the regular structure of palisade endings. Palisade endings are formed by nerve fibers that come from the muscle and extend into the tendon. There, axons make a U-shaped turn to approach the muscle–tendon junction, and, by further branching, they establish synaptophysin-positive terminal varicosities at the level of the muscle fiber tips and the tendon (Figs. 2A, 2B).

### Palisade Endings Following Unilateral Visual Deprivation

We tested whether unilateral visual deprivation affected the development of palisade endings at P30. The contralateral eye had visual experience and served as control (Fig. 1A). On the control sides, palisade endings were not developed at the age of 30 days in the superior rectus and lateral rectus muscles. Specifically, the nerve fibers forming palisade endings stopped at the level of the muscle without extending into the tendon (data not shown). Only in the inferior rectus and medial rectus muscle were palisade endings present. They were more developed in the medial rectus than in the inferior rectus muscle, as indicated by a higher degree of axonal branching and synaptophysin-positive terminal varicosities (Fig. 3A for the inferior rectus; Figs. 3B, 3C for the medial rectus). These observations are in agreement with our previous study,^19^ where we showed that palisade endings develop following a muscle-specific time course and develop later in the superior rectus and lateral rectus muscles than in the inferior rectus and medial rectus muscle.

Unilateral visual deprivation did not affect palisade ending development. Specifically, as in the control side, palisade endings were absent in the superior rectus and lateral rectus muscles and were only present in the inferior rectus and medial rectus muscle of the covered eye. Analogous to the control side, palisades were more complex in the medial rectus than in the inferior rectus muscle (Fig. 3A’ for the inferior rectus muscle; Figs. 3B’, 3C’ for the medial rectus muscle).

To test if unilateral visual deprivation affected the number of palisade endings per muscle, we quantified palisade endings in the covered and uncovered eye (n = 3 for each group at 30 days). Quantitative analyses exhibited no side difference, and on both sides many more palisade endings were present in the medial rectus than in the inferior rectus muscle (Fig. 3D, Table).

**Table.** Quantification of the Number of Palisade Endings in Each Experimental Situation. Data Are Shown as Mean and SEM

		Number of Palisade Endings				
		Unilateral Light Deprivation (P30)		Bilateral Light Deprivation (P45)		
Muscle	N	Control Left Eye	Covered Right Eye	Control Eye	Covered Left Eye	Covered Right Eye
SR	3	0.0 ± 0.0	0.0 ± 0.0	15.0 ± 1.5	14.7 ± 0.9	13.3 ± 0.3
LR	3	0.0 ± 0.0	0.0 ± 0.0	16.0 ± 0.6	16.3 ± 1.5	15.7 ± 1.2
IR	3	28.3 ± 0.9	29.3 ± 1.5	35.0 ± 1.2	35.7 ± 2.3	38.0 ± 1.7
MR	3	106.7 ± 3.8	108.7 ± 5.8	104.0 ± 4.4	105.0 ± 2.6	106.3 ± 5.2
		**Unilateral BoNA-Injection (P45)**		**Unilateral BoNA-Injection (P95)**		
**Muscle**	**N**	**Control Side**	**BoNT-A Side**	**Control Side**	**BoNT-A Side**
SR	3	12.0 ± 1.2^*^	0.0 ± 0.0	24.3 ± 3.0^*^	0.0 ± 0.0	
LR	3	14.0 ± 0.6^*^	0.0 ± 0.0	24.0 ± 5.0^*^	0.0 ± 0.0	
IR	3	34.7 ± 0.9^*^	23.3 ± 3.8	53.7 ± 6.9^*^	28.7 ± 4.8	
MR	3	108.7 ± 4.8^*^	58.7 ± 5.2	102.7 ± 2.6^*^	49.3 ± 6.9	

Asterisks (*) indicate significant differences between control side and BoNT-A side at P45 and P95.

We further evaluated if unilateral visual deprivation affected terminal varicosities of palisade endings by determining the area covered by synaptophysin signals of palisade endings in the covered and uncovered eye (n = 3 for each group). Analyses in palisade endings of the medial rectus muscle showed that there was no difference between sides with respect to synaptophysin (Fig. 3E). Because our unilateral visual deprivation experiments did not produce changes in palisade ending development, we used a double-eye-covered paradigm and longer deprivation time span (150%) to ensure maximal effects, if any.

### Palisade Endings Following Bilateral Visual Deprivation

At P45, in animals with bilateral visual deprivation (Fig. 1B), palisade endings in the rectus muscles of both eyes were analyzed and compared with palisade endings from an age-matched sibling (control), which grew up in the normal lighted environment of the animal house as a littermate. In the control, we observed palisade endings in all rectus muscles, and this confirmed our previous study^19^ that palisade endings are regularly present 45 days after birth in all four rectus muscles (Figs. 4A–4D). Because of the heterochronic developmental sequence,^19^ palisade endings were less developed in the superior rectus and lateral rectus muscles, and they had fewer axonal branches and fewer terminal varicosities, as indicated by low levels of synaptophysin labeling (Fig. 4A for the superior rectus muscle; Fig. 4B for the lateral rectus muscle). Palisade endings were more developed in the inferior rectus muscle and had a higher degree of axonal branching and synaptophysin-positive terminal varicosities (Fig. 4C). Exclusively, in the medial rectus muscle, palisade endings exhibited almost adult-like characteristics at the age of P45 (Fig. 4D).

In animals with bilateral visual deprivation (Fig. 1B), palisade endings were present in all rectus muscles of both eyes at P45. In the same muscles from both covered eyes, palisade endings were structurally similar to each other (Figs. 4A’, 4A”, 4B’, 4B”, 4C’, 4C”, 4D’, 4D”). Furthermore, palisade endings in visually deprived animals and the age-matched control were structurally indistinguishable. As in the control, palisade endings in visually deprived animals developed in a heterochronic sequence. Specifically, they were less developed in the superior and lateral rectus muscle (Figs. 4A’, 4A”, 4B’, 4B”), more developed in the inferior rectus muscle (Figs. 4C’, 4C”), and almost mature in the medial rectus muscle (Figs. 4D’, 4D”).

We quantified palisade endings in visually deprived and control animals (*n* = 3 for each group). One control muscle came from the present study and two other age-matched controls from our previous study.^19^ In both animal groups, the number of palisade endings varied among the rectus muscles. That is, the highest number of palisade endings was counted in the medial rectus muscle (Figs. 4D, 4D’, 4D”, 4E; Table), and the lowest number in the lateral rectus and the superior rectus muscles (Figs. 4A, 4B, 4A’, 4B’, 4A”, 4B”, 4E; Table). Values for the inferior rectus muscle were in between (Figs. 4C, 4C’, 4C”, 4E; Table). No significant difference in the absolute number of palisade endings per muscle was observed between controls and visually deprived animals (Fig. 4E, Table).

We determined the area covered by synaptophysin signals in palisade endings of controls and visually deprived cats (*n* = 3 for each group). Analyses of palisade endings of the left medial rectus muscle in controls and visually deprived animals showed that there was no side difference in synaptophysin values (Fig. 4F). Taken together, our results show that following unilateral or bilateral visual deprivation, palisade endings did not differ qualitatively and quantitatively from palisade endings of controls.

### Unilateral BoNT-A Injection

**Figure 5.**
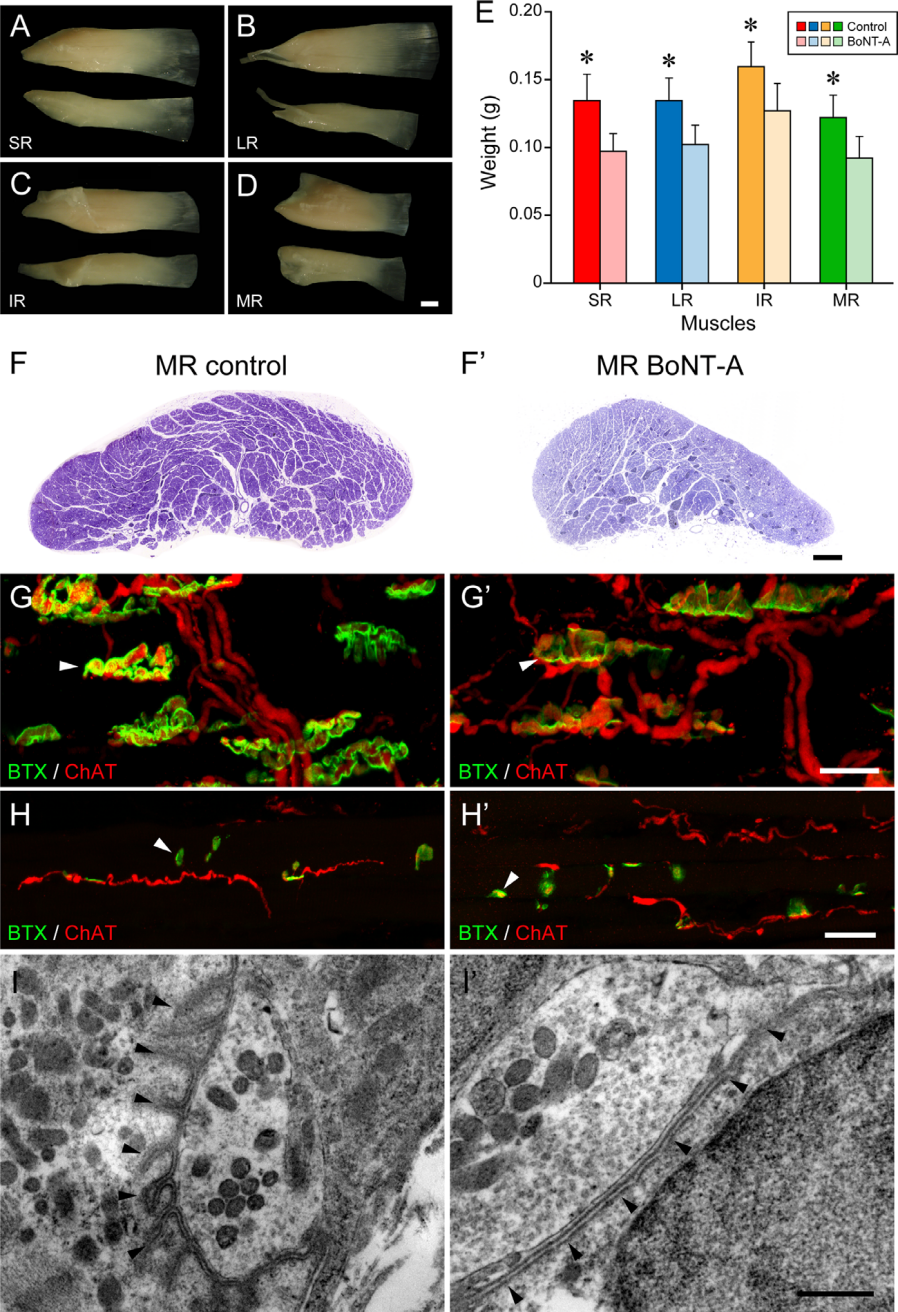
Changes in muscles and innervation following BoNT-A injection. Image data are from P95 animals. (**A**–**D**) The rectus muscles on the control side (upper example) and injected side (lower example). The superior rectus (SR) muscle (**A**), lateral rectus (LR) muscle (**B**), inferior rectus (IR) muscle (**C**), and medial rectus (MR) muscle (**D**) are thinner on the injected side. (**E**) Bar chart illustrating that rectus muscles on the injected side are of lower weight. Asterisk (*) indicates statistically significant weight differences. Mean ± SEM for weight of control and BoNT-A side: SR, 0.135 ± 0.019/0.098 ± 0.013 (paired *t*-test, *t*(3) = −4.392, *P* = 0.022); LR, 0.138 ± 0.014/0.100 ± 0.012 (paired *t*-test, *t*(3) = −4.392, *P* = 0.022); IR, 0.160 ± 0.018/0.128 ± 0.020 (paired *t*-test, *t*(3) = −13.000, *P* ≤ 0.001); MR, 0.123 ± 0.017/0.093 ± 0.016 (paired *t*-test, *t*(3) = −3.286, *P* = 0.046). (**F**, **F’**) Medial rectus muscles from the control side (**F**) and injected side (**F’**) showing that the cross-sectional area is smaller at the injected side. (**G**, **G’**, **H**, **H’**) Immunofluorescence staining of nerve fibers labeled with anti-ChAT (*red*) and motor terminals (en plaque and en grappe terminals) with α-bungarotoxin (BTX, *green*). On the control side, en plaque motor terminals (*white arrowhead*) on singly innervated muscle fibers are more complex (**G**) than on the injected side (**G’**). En grappe terminals (*white arrowhead*) are structurally indistinguishable on the control and injected sides (**H**, **H’**). (**I**, **’I’**) Fine structural features of en plaque motor terminals on the control side (**I**) and injected side (**I’**). Motor terminals on the control side exhibit subsynaptic folding (**I**, *black arrowheads*), whereas on the injected side the subsynaptic membrane is smooth (**I’**, *black arrowheads*). *Scale bars*: 1 mm in **D** (for **A**, **B**, **C**, **D**), 100 µm in **F’** (for **F**, **F’**), 20 µm in **G’** (for **G**, **G’**), 25 µm in **H’** (for **H**, **H’**), and 0.5 µm in **I’** (for **I** and **I’**).

Around birth (P0), a single BoNT-A injection was made into the retrobulbar space of the left eye (Fig. 1C). The uninjected contralateral eye served as control. The survival time of the animals was 45 (three cats) and 95 days (four cats). Following BoNT-A injection, we observed that in both P45 and P95 animals the rectus muscles on the injected side were thinner and of smaller cross-sectional area (Figs. 5A–5D, 5F, 5F’). Muscle weight measurements were performed in P95 animals, and all muscles on the injected side were of significantly lower weight than those of the control non-injected eye (Fig. 5E). We examined en plaque motor terminals on singly innervated muscle fibers and en grappe motor terminals on multiply innervated muscle fibers on the control and injected sides. Based on ChAT and α-bungarotoxin staining, we observed that the en plaque motor terminals were less elaborate on the injected side (Figs. 5G, 5G’), whereas en grappe motor terminals exhibited no side difference (Figs. 5H, 5H’). It is possible that structural changes in the en grappe terminals were not so apparent because of the small size of these terminals. Ultrastructural analyses revealed that en plaque motor terminals did not display folding of the postsynaptic membrane on the injected side (Figs. 5I, 5I’). By periodic visual inspection, we observed misalignment of the animal’s eyes for the entire period of the BoNT-A experiment (45 and 95 days). Additionally, at 90 days, we could not see any eye movements, such as saccades (even slow ones), or the fast and slow phases of the vestibular nystagmus when the animal was rotated. This confirmed the long-lasting paralyzing effect of BoNT-A and suggests that BoNT-A remains active at the terminal side, impeding neuromuscular transmission for at least 95 days. Altogether, these data are in accordance with the literature,^26^^,^^30^ as indicative of the neurotoxin effectiveness.

### Palisade Endings Following Unilateral BoNT-A Injection

Forty-five and 95 days after unilateral BoNT-A administration (Fig. 1C), palisade endings were analyzed on the control and injected sides.

#### Palisade Endings in P45 Animals

**Figure 6.**
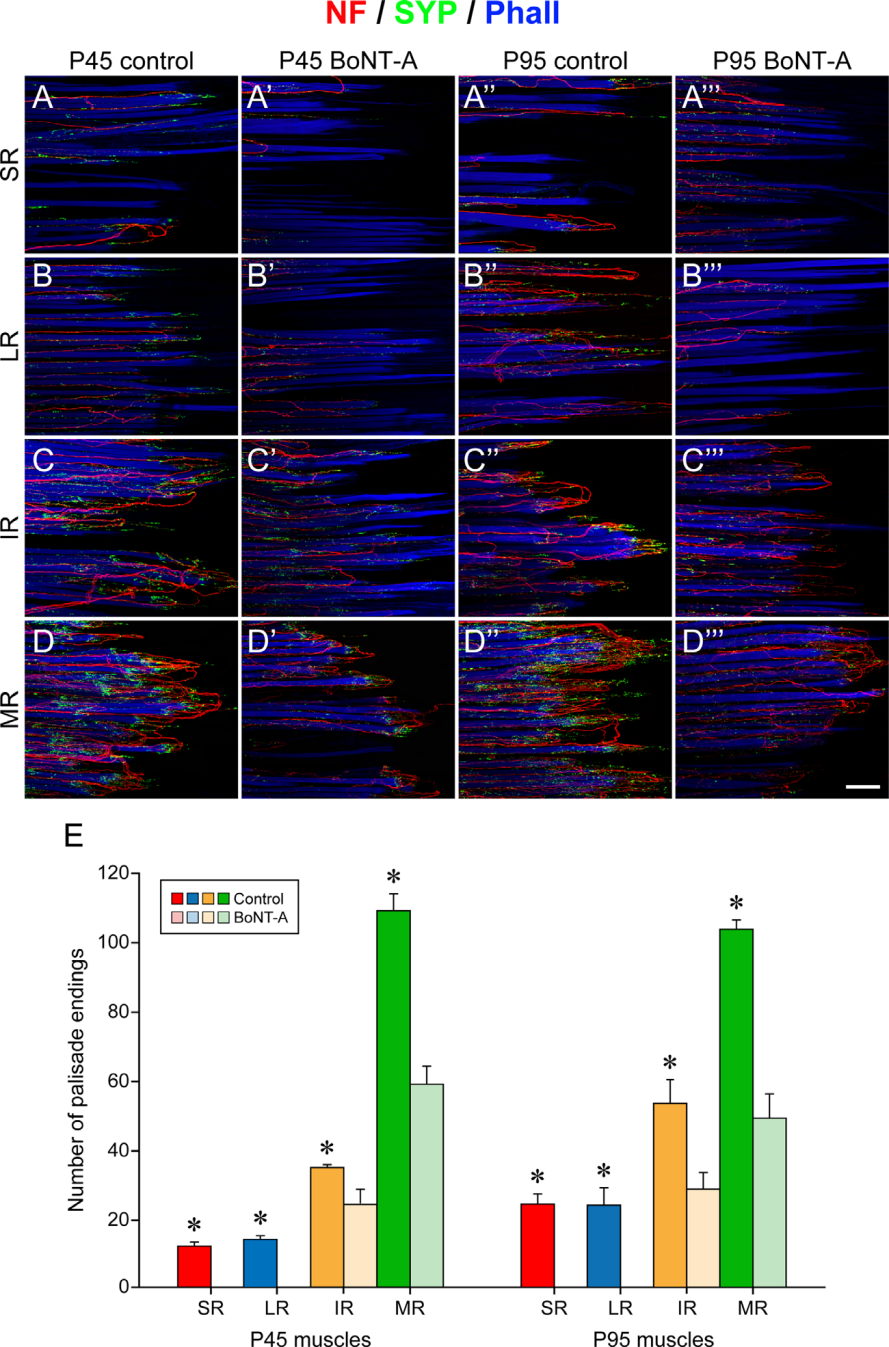
Structural features and number of palisade endings on the control and injected sides. (**A**–**D**, **A’**–**D’**, **A”**–**D”**, **A’’’**–**D’’’**) Three-dimensional projection of palisade endings in neurofilament (NF, red), synaptophysin (SYP, *green*), and phalloidin (Phall, *blue*) labeling. Palisade endings are visualized in P45 and P95 animals on the control side (P45, **A**–**D**; P95, **A”**–**D”**) and the BoNT-A injected side (P45, **A’**–**D’**; P95, **A’’’**–**D’’’**). On the control side of P45 animals, palisade endings are present in all rectus muscles. They are less developed in the superior rectus (SR) muscle (**A**) and lateral rectus (LR) muscle (**B**), more developed in the inferior rectus (IR) muscle (**C**), and almost adult-like in the medial rectus (MR) muscle (**D**). On the injected side of P45 animals, palisade endings are absent in the SR and LR muscles (**A’**, **B’**), and nerve fibers stop at the level of the muscle. Palisade endings are present in the IR and MR muscle (**C’**, **D’**), but they are simpler and express less synaptophysin than palisades in the corresponding control muscles (compare **C** with **C’** and **D** with **D’**). At the age of P95, palisade endings have an adult-like quality in all rectus muscles on the control side: SR muscle (**A”**), LR muscle (**B”**), IR muscle (**C”**), and MR muscle (**D”**). On the injected side of P95 animals, palisade endings are absent in the SR muscle (**A’’’**) and LR muscle (**B’’’**), and nerve fibers stop at the muscle level. Palisade endings are present in the IR muscle (**C’’’**) and MR muscle (**D’’’**), but they are smaller and simpler. They express less synaptophysin than palisades in control muscles (compare **C”** with **C’’’** and **D”** with **D’’’**). (**E**) Bar chart showing the number of palisade endings for each rectus muscle on the control and injected sides in P45 and P95 animals. In both age groups, there are significant differences (as indicated by asterisks) in the palisade ending number between the control and injected sides. Mean ± SEM for palisades in all rectus muscles of the control/BoNT-A injected eye are shown. Counts at age P45: SR, 12.0 ± 1.2/0.0 ± 0.0 (*t*-test, *t*(4) = 10.392, *P* ≤ 0.001); LR, 14.0 ± 0.6/0.0 ± 0.0 (*t*-test, *t*(4) = 24.249, *P* ≤ 0.001); IR, right control eye, 34.7 ± 0.9/23.3 ± 3.8 (*t*-test, *t*(4) = 2.874, *P* = 0.045); MR, 108.7 ± 4.8/58.7 ± 5.2 (*t*-test, *t*(4) = 7.032, *P* = 0.002). Counts at age P95: SR, 24.3 ± 3.0/0.0 ± 0.0 (*t*-test, *t*(4) = 8.213, *P* = 0.001); LR, 24.0 ± 5.0/0.0 ± 0.0 (*t*-test, *t*(4) = 4.768, *P* = 0.009); IR, 53.7 ± 6.9/28.7 ± 4.8 (*t*-test, *t*(4) = 2.969, *P* = 0.041); MR, 102.7 ± 2.6/49.3 ± 6.9 (*t*-test, *t*(4) = 7.243, *P* = 0.002). *Scale bars*: 100 µm in **D’’’** (for all images and **D’’’**).

**Figure 7.**
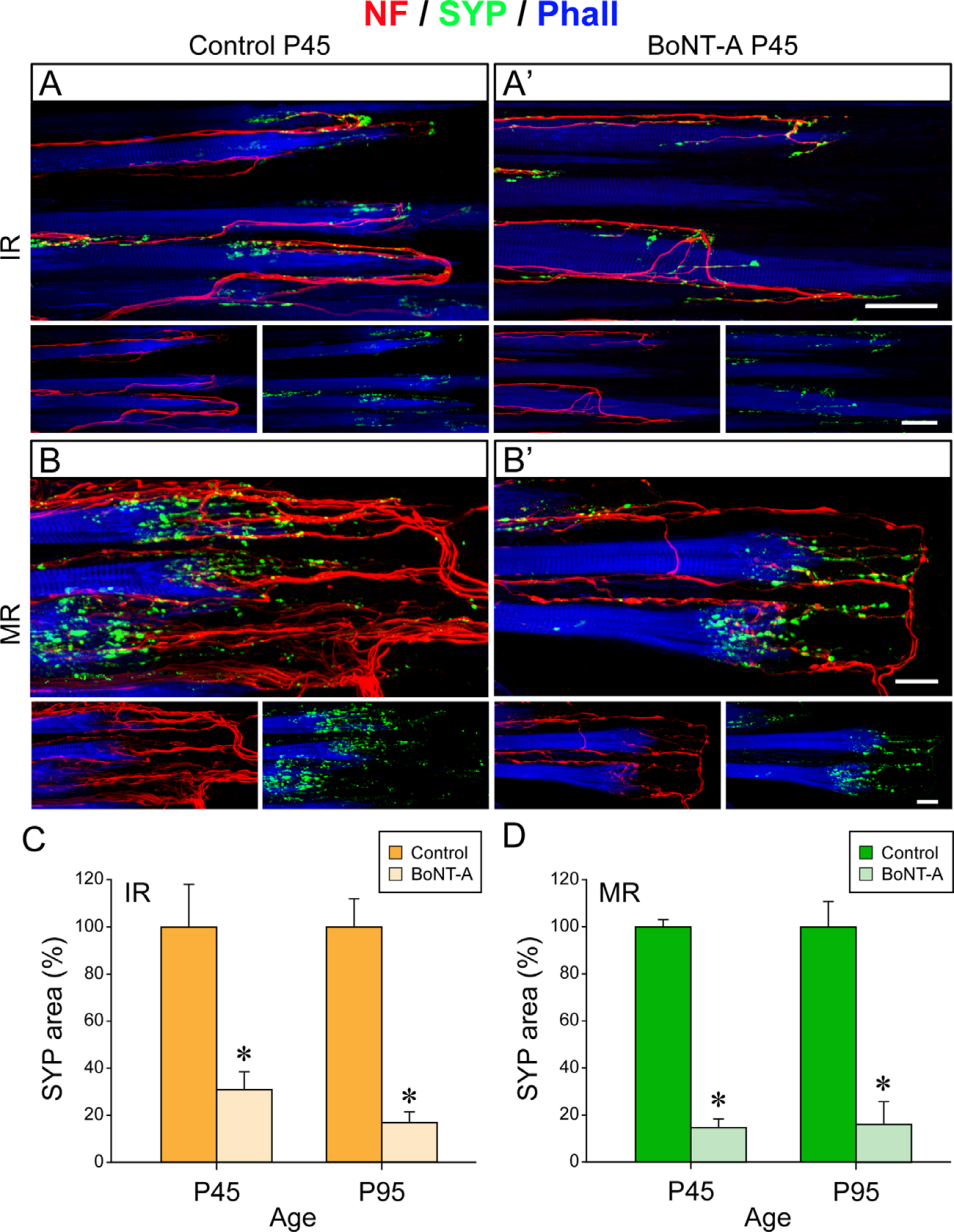
Structural changes of palisade endings in P45 animals following BoNT-A injection. (**A**, **A’**, **B**, **B’**) Three-dimensional projection of palisade endings in neurofilament (NF, *red*), synaptophysin (SYP, *green*), and phalloidin (Phall, *blue*) labeling. Palisade endings are visualized in the inferior rectus (IR) and medial rectus (MR) muscles on the control side (**A**, **B**) and injected side (**A’**, **B’**). Palisade endings in control muscles (IR, **A**; MR, **B**) have a higher degree of axonal branching and more terminal varicosities compared to palisade endings on the injected side (IR, **A’**; MR, **B’**). Generally, palisade endings are simpler in the IR muscle (**A**, **A’**) than in the MR muscle (**B**, **B’**)¸ irrespective of control or injected side. (**C**, **D**) Bar charts showing the area of synaptophysin-labeled boutons in palisade endings on the control and injected side at age P45 and P95. In palisade endings of both the IR muscle (**C**) and the MR muscle (**D**), the area of synaptophysin-labeled boutons is significantly higher (asterisk) on the control than injected side. Mean ± SEM of the area of synaptophysin-labeled boutons at the control/injected side: P45-IR (**C**), 100.0 ± 20.7/31.8 ± 3.9 (*t*-test, *t*(4) = 3.243, *P* = 0.032); P95-IR (**C**), 100.0 ± 11.7/16.8 ± 6.4 (*t*-test, *t*(4) = 6.241, *P* = 0.003); P45-MR (**D**), 100.0 ± 2.4/15.3 ± 2.8 (*t*-test, *t*(4) = 22.832, *P* ≤ 0.001); P95-MR (**D**), 100.0 ± 13.2/16.6 ± 8.8 (*t*-test, *t*(4) = 5.260, *P* = 0.006). *Scale bars*: 50 µm in **A’** (for **A**, **A’**) and 20 µm in **B’** (for **B**, **B’**).

On the control side, palisade endings were present in all rectus muscles, and, with respect to qualitative features, they were indistinguishable from palisade endings of other P45 animals (Figs. 6A–6D). In contrast, after BoNT-A treatment, palisade endings were absent in the superior rectus and lateral rectus muscles of P45 animals; instead, nerve fibers terminated at the level of the muscle at different distances from the muscle–tendon junction (Figs. 6A’, 6B’). Only in the inferior rectus and medial rectus muscle were any palisade endings present following BoNT-A injection (Figs. 6C’, 6D’). They were, however, simpler in the BoNT-A–treated inferior rectus muscle, and the nerve fibers forming palisade endings did not penetrate far into the tendon. Instead, they turned back at the level of the muscle fiber tip (Fig. 6C’, low magnification; Fig. 7A’, high magnification). When comparing palisade endings in the inferior rectus and medial rectus muscle on the control and injected sides, we observed that the palisade endings of the injected side exhibited a rudimentary phenotype. In particular, the palisade endings on the injected side showed a reduction in size, axonal branching, and synaptophysin-positive terminal varicosity numbers (Figs. 7A, 7B, control side; Figs. 7A’, 7B’, injected side).

#### Palisade Endings in P95 Animals

**Figure 8.**
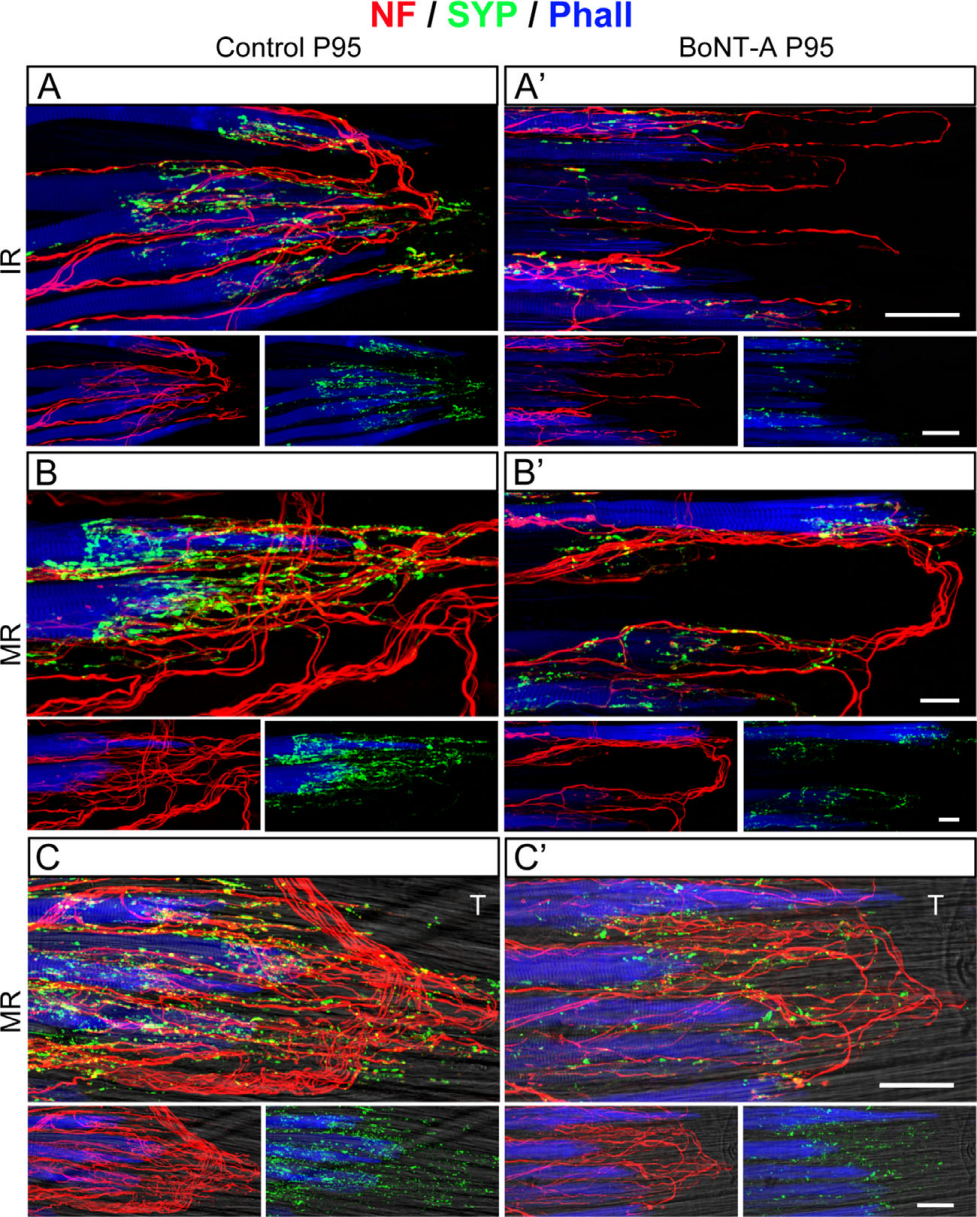
Changes in palisade endings of P95 animals following BoNT-A injection. (**A**, **A’**, **B**, **B’**, **C**, **C’**) Three-dimensional projections of CLSM images showing palisade endings in anti-neurofilament (NF, *red*), anti-synaptophysin (SYP, *green*), and phalloidin (Phall, *blue*) labeling. The tendon not visible in **A**, **A’**, **B**, **B’** is visualized in **C** and **C’** by adding a brightfield view. At the age of P95, palisade endings are adult-like in the inferior rectus (IR) muscle (**A**) and medial rectus (MR) muscle (**B**, **C**) of the control side. Palisade endings exhibit complex axonal branching and numerous synaptophysin-positive terminal varicosities. Terminal varicosities are at the level of the muscle fiber and the tendon (T), which is visualized in **C**. On the injected side (**A’**, **B’**, **C’**), palisade endings in the IR (**A’**) and MR muscle (**B’**, **C’**) are simpler. They exhibit less axonal branching and less synaptophysin-positive terminal varicosities at the level of the muscle fiber and the tendon (**C’**). Additionally, on the injected side, palisade endings are simpler in the IR muscle (**A’**) than in the medial rectus muscle (**B’**, **C’**). In the lower part of **A**, **A’**, **B**, **B’**, **C**, and **C’**, the color channels are separated to illustrate either only neurofilament (in *red*) or synaptophysin (in *green*) in addition to phalloidin. *Scale bars*: 50 µm in **A’** and **C’** (for **A**, **A’**, **C**, **C’**) and 20 µm in **B** (for **B**, **B’**).

At the age of P95, palisade endings exhibited adult-like aspects in all rectus muscles on the control side (Figs. 6A”–6D”), which is in agreement with our previous study.^19^ In contrast, palisade endings were absent in the superior rectus and lateral rectus muscles on the injected side, and the nerve fibers stopped at the level of the muscle, although closer to the muscle–tendon junction than in P45 animals (Figs. 6A’’’, 6B’’’). In the BoNT-A–treated inferior rectus and medial rectus muscles, palisade endings were present, and, analogous to P45 animals, they were simpler in the inferior rectus muscles (Figs. 6C’’’, 6D’’’, low magnification; Figs. 8A’, 8B’, 8C’, high magnification). Structurally, palisade endings in the BoNT-A–treated inferior rectus and medial rectus muscle did not achieve the same quality of development as in the corresponding control muscles. Specifically, BoNT-A–treated palisade endings were smaller and exhibited fewer axonal branches and lower levels of synaptophysin (Figs. 8A, 8B, 8C, control side; Figs. 8A’, 8B’, 8C’, injected side).

Quantitative analyses showed that following BoNT-A treatment the number of palisade endings was reduced (Fig. 6E, Table). Specifically, on the injected side, palisade endings were absent in the superior rectus and lateral rectus muscles of P45 and P95 animals, whereas in controls around 15 palisade endings were counted at the age of P45 and 25 palisade endings at P95 (Fig. 6E, Table). In the BoNT-A–treated medial rectus muscle, the number of palisade endings was about 50% less in both P45 and P95 animals (Fig. 6E, Table), and the same difference was observed in the inferior rectus muscles at P95. Less difference was observed between the inferior rectus muscles of P45 animals (Fig. 6E, P45 muscles).

Measurements of the area covered by synaptophysin-labeled terminals were done in palisade endings of the inferior rectus and medial rectus muscles. At the ages of both P45 and P95, the synaptophysin coverage was reduced in palisade endings on the injected side (Fig. 7C, inferior rectus muscle at age P45 and P95; Fig. 7D, medial rectus muscle at P45 and P95).

#### GAP43 Expression in Palisade Endings

GAP43 is a nervous tissue specific protein that is expressed during neuronal development and neuronal regeneration.^31^ In palisade endings, the GAP43 expression correlates with the degree of maturity.^19^ It is high when palisades are simple and immature and low when they are adult-like.^19^ Because the present findings demonstrated that palisade endings were rudimentary under the influence of BoNT-A, we tested for GAP43 expression. Distal myotendons of the control and injected sides of a P95 cat were immunolabeled with anti-neurofilament/anti-GAP43 along with phalloidin. To compare the GAP43 expression in palisade endings of the P95 cat and younger animals, we immunolabeled a medial rectus muscle of a P22 cat from a previous study.^19^ This P22 cat did not receive BoNT-A injection.

**Figure 9.**
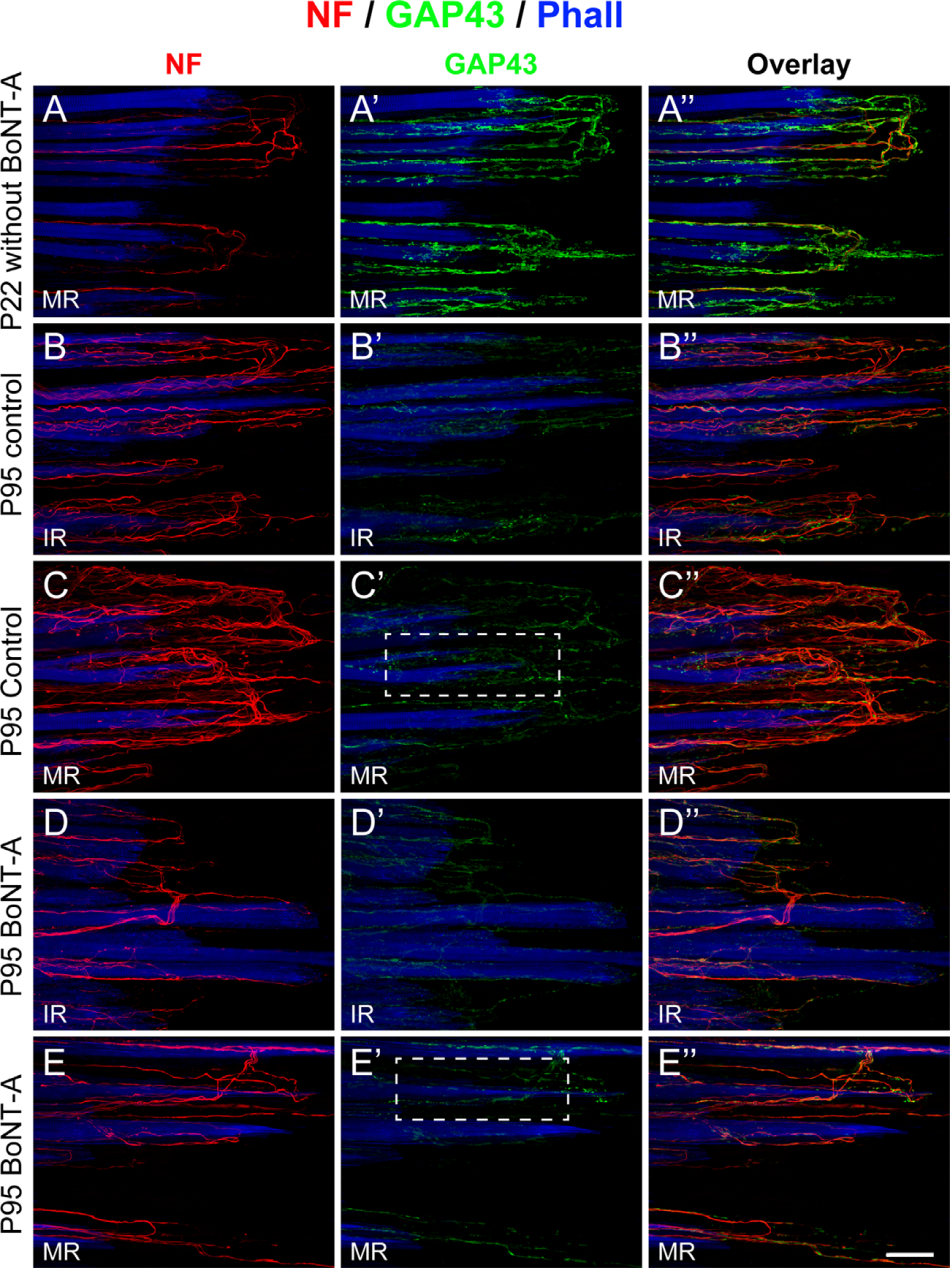
Expression of the developmental protein GAP43 in palisade endings of a P22 cat without BoNT-A treatment and palisade endings of a P95 cat following BoNT-A injection. (**A**–**A”**, **B**–**B”**, **C**–**C”**, **D**–**D”**, **E**–**E”**) Three-dimensional projections of CLSM images showing palisade endings labeled with anti-neurofilament (NF), anti-GAP43, and phalloidin (Phall). At the age of P22, palisade endings in the medial rectus (MR) muscle (**A**–**A”**) are not fully developed and they express high levels of GAP43 (**A’**). At the age of P95, palisade endings of the inferior rectus (IR) muscle (**B**–**B”**) and MR muscle (**C**–**C”**) of the control side are adult-like and exhibit low levels of GAP43 (**A’**, **B’**). On the injected side of the P95 cat, palisades in the IR muscle (**D**–**D”**) and medial rectus muscle (**E**–**E”**) are rudimentary. Analogous to the control side, palisade endings on the injected side exhibit low levels of GAP43 (**D’**, **E’**). The overlays of neurofilament and GAP43 staining are shown in **A”**, **B”**, **C”**, **D”,** and **E”**. *Scale bars*: 50 µm in **E”** (for all other images and **E”**). For insets in **C’** and **E’**, see the legend for Figure 10.

In the P22 cat, palisade endings of the medial rectus muscle were immature, and they exhibited strong GAP43 expression (Figs. 9A–9A”). In the P95 cat, palisade endings were adult-like on the control side, and they expressed low levels of GAP43. Data are shown for palisade endings of the inferior rectus and medial rectus muscle (Figs. 9B–9B”, 9C–9C”). The high GAP43 expression in P22 palisade endings versus the low GAP43 expression in P95 palisade endings is consistent with our prior findings on palisade ending development.^19^ On the BoNT-A side of the P95 cat, palisade endings of the inferior rectus and medial rectus muscles were rudimentary. Analogous to the control side, the GAP43 expression was low in palisade endings of the injected side (Figs. 9D–9D”, 9E–9E”).

**Figure 10.**
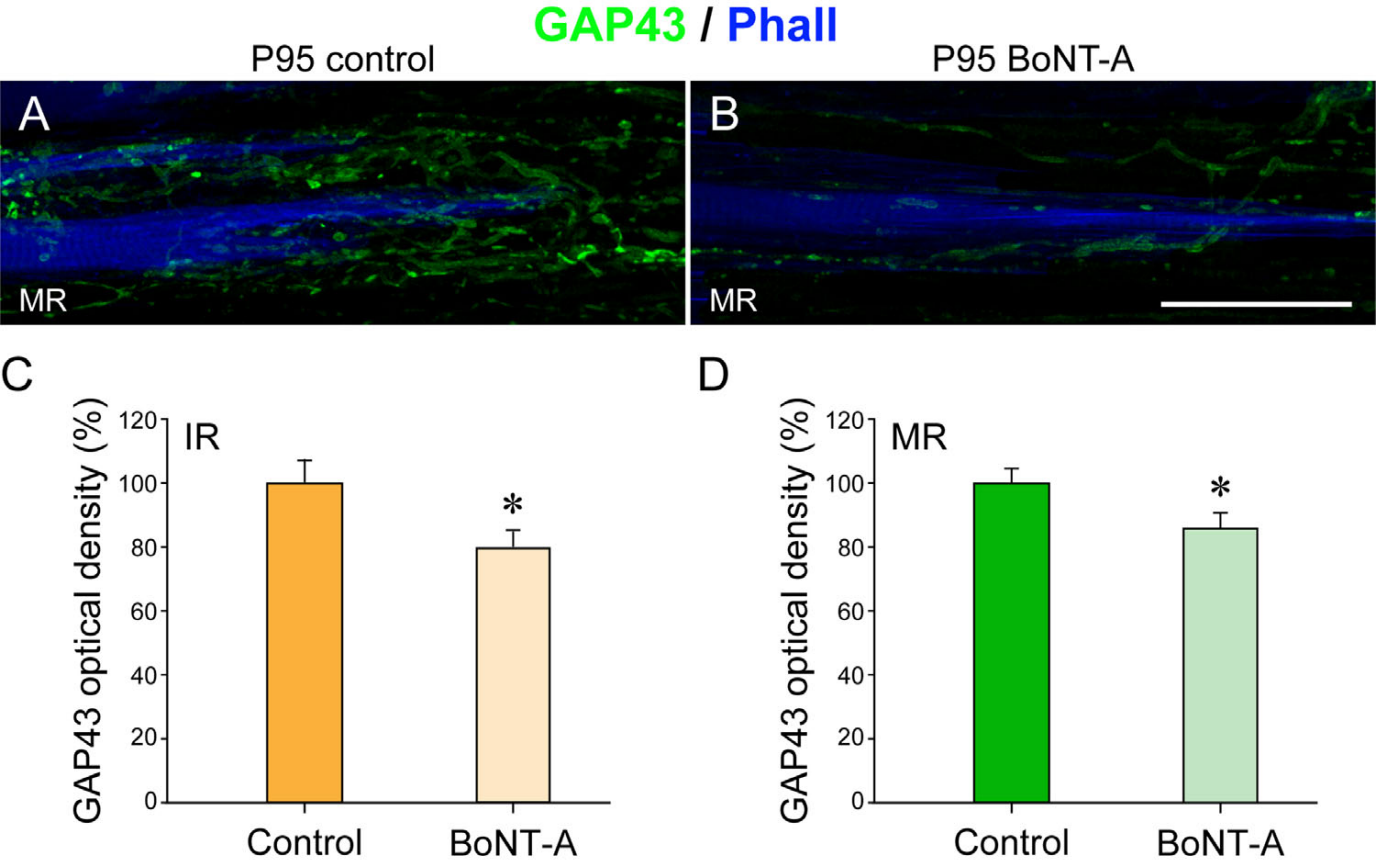
Quantification of GAP43 in palisade endings of a BoNT-A–injected P95 cat. (**A**, **B**) Higher magnification three-dimensional projections of CLSM images showing palisade endings of the medial rectus on the control and injected side (insets in Figs. 9C’, 9E’). At the age of P95, the GAP43 signal is higher on the control side (**A**) than on the injected side (**B**) in the medial rectus. (**C**, **D**) Bar chart showing that the optical density of the GAP43 signal measured in individual palisade endings of the inferior rectus (IR) muscle (**C**) and medial rectus (MR) muscle (**D**) is significantly lower (asterisk) on the BoNT-A side than on the control side. Mean ± SEM: 100.0 ± 6.7 for IR control side, 80.6 ± 6.3 for IR BoNT-A (*t*-test, *t*(98) = 2.095, *P* = 0.039); 100.0 ± 4.1 for the MR control side, 86.4 ± 4.3 for MR BoNT-A side (*t*-test, *t*(98) = 2.296, *P* = 0.024). *Scale bar*: 50 µm in **B** (for **A** and **B**).

We quantified the GAP43 intensity in palisade endings of the P95 animal on the control and injected sides. This analysis indicated that the GAP43 signal in palisade endings of the inferior rectus and medial rectus muscles was significantly lower on the injected side than even the low values of the adult-like (P95) control side (Figs. 10C, 10D). This difference can be seen in the high-magnification views (Figs. 10A, 10B).

#### En Grappe Motor Terminals Associated with Palisade Endings

**Figure 11.**
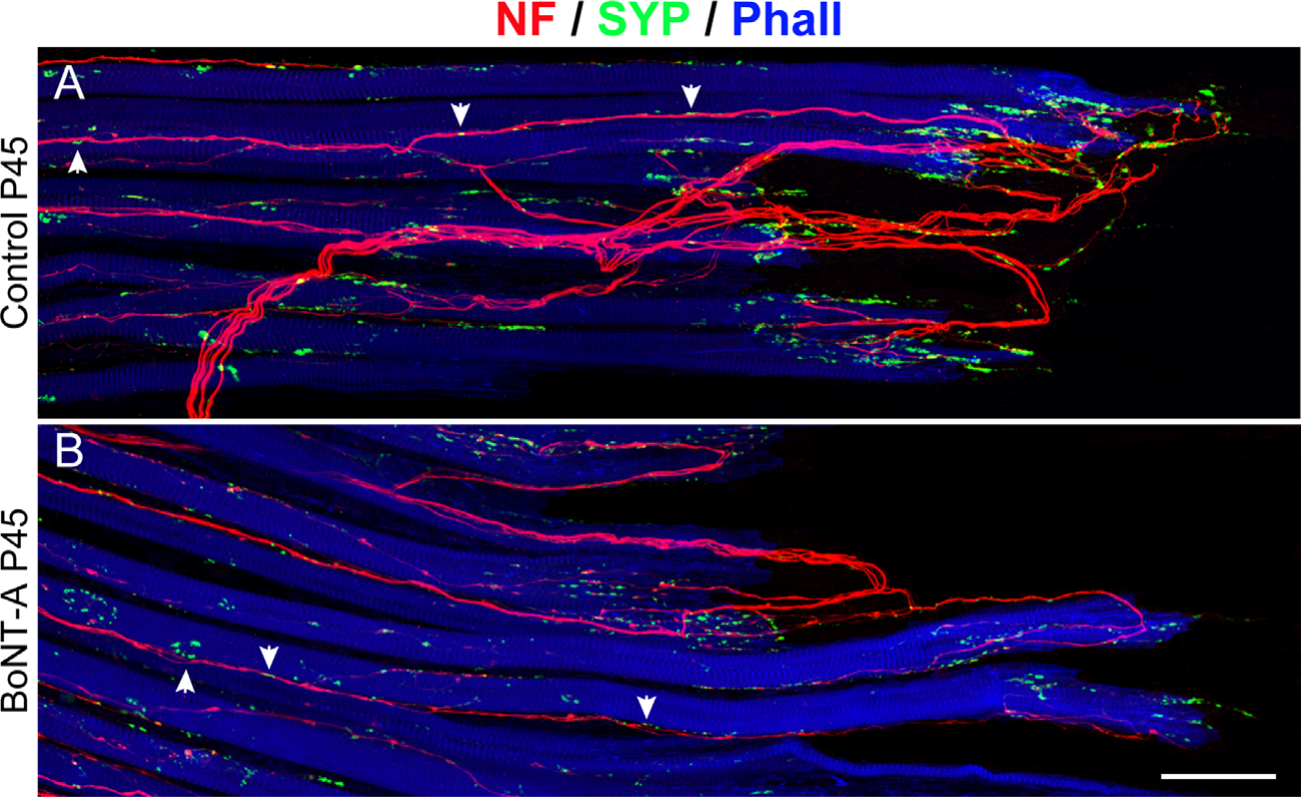
Structure of palisade endings and associated en grappe motor terminals in control and BoNT-A–treated P45 animals. (**A**, **B**) Stitched CLSM images of palisade endings stained with anti-neurofilament (NF, *red*), anti-synaptophysin (SYP, *green*), and phalloidin (Phall, *blue*). In the control animals, palisade endings are complex (**A**), but they are rudimentary following BoNT-A treatment. En grappe motor terminals (*arrows*) associated with palisade endings are structurally similar on the control side (**A**) and BoNT-A injected side (**B**). *Scale bar*: 50 µm in **B** (for **A** and **B**).

There is indication from the literature that palisade endings are expansions of motoneuron axons that establish multiple en grappe motor terminals alongside muscle fibers.^16^^,^^18^ Motoneurons that establish multiple motor terminals are termed MIF motoneurons as opposed to SIF motoneurons that establish a single motor endplate.^32^ We looked for palisade endings associated with en grappe motor terminals on the control and injected sides. In both P45 and P95 animals, we observed that en grappe terminals associated with palisade endings were similar in appearance on the control and injected sides (Figs. 11A, 11B).

Taken together, our results demonstrate that, following BoNT-A injection, palisade endings exhibited distinct qualitative and quantitative changes. Importantly, analogous to the control side, a muscle-specific time course of palisade ending development and muscle-specific density were observed after BoNT-A administration (i.e., palisade endings were further developed and more numerous in the medial rectus than in the other rectus muscles). The expression of proteins related to neuronal development was significantly lower on the injected side. En grappe motor terminals associated with palisade endings exhibited no side difference.

## Discussion

**Figure 12.**
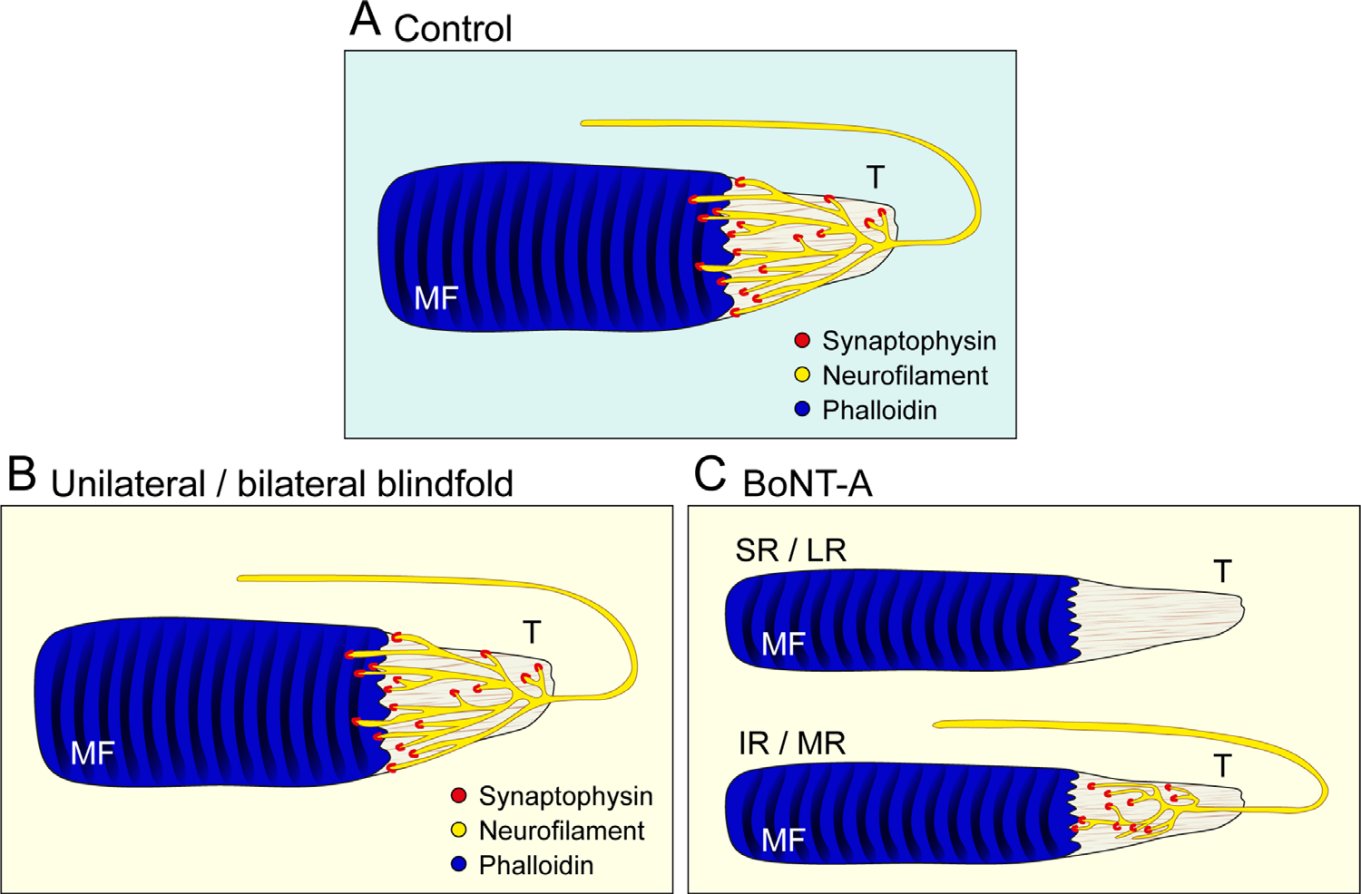
Schematic diagram summarizing the results of the present study. (**A**) Axons forming palisade endings in a control situation establish synaptophysin-positive terminal varicosities at the tendon (T) level and muscle fiber (MF) tips. (**B**) Palisade endings following bilateral or unilateral visual deprivation do not differ from the control situation. (**C**) After BoNT-A injection, palisade endings are absent in the superior rectus (SR) and lateral rectus (LR) muscle, and palisade endings with simple phenotype are present in the inferior rectus (IR) muscle and medial rectus (MR) muscle. The eye muscles and consequently their muscle fibers are thinner after single BoNT-A administration.

In cat, a frontal-eyed species, palisade endings develop in the first 3 months of life^19^ and at the same time as visuomotor behavior develops.^33^ The maturation of visuomotor coordination requires visual experience and visually guided eye movements.^20^ Because palisade endings and visuomotor coordination develop simultaneously, it is possible that vision and/or eye movements might be relevant for palisade ending maturation, as well. To test this, we performed visual deprivation and eye immobilization in newborn kittens. We demonstrated that, when vision is prevented, palisade endings develop normally, but when eye movements are prevented palisade ending development is altered and the number of palisade endings is reduced (Fig. 12). These findings suggest that eye movements, but not vision, play an essential role for the postnatal maturation of palisade endings.

### Vision Does Not Drive Palisade Ending Development

Visual experience after birth is important for the maturation of the visual sensory system.^34^ Additionally, there is evidence that vision in the early postnatal period is crucial for development of the oculomotor system.^35^^–^^38^ Specifically, bilateral and unilateral visual deprivation for a period of 45 days after birth in rats disrupts the expression of EOM-specific myosin, although these effects are more severe after bilateral visual deprivation.^35^ In the same animal species, unilateral blindness after birth reduces the diameter of the trochlear nerve, but changes are more severe in larger than smaller axons.^38^ Unilateral visual deprivation in kittens leads to a reduction of muscle fiber size, capillary density, and contractile forces in EOMs.^36^^,^^37^ Vision is associated with eye movements, and it has been suggested that loss of vision reduces the demand for visually guided eye movements.^35^^,^^36^ This drop in visuomotor activity prevents EOMs and cranial nerves innervating EOMs from reaching the adult phenotype.^35^^–^^38^ With respect to palisade ending development, we did not observe that loss of vision had any effect at the structural level. Specifically, following unilateral and bilateral visual deprivation, palisade endings in the rectus muscles were qualitatively and quantitatively equivalent to palisade endings of age-matched controls. Moreover, the muscle-specific time course of palisade ending development was the same as in controls. Because palisade endings developed normally following visual deprivation, our findings must be interpreted to indicate that vision and visually guided eye movements do not play a role in palisade ending development. It is important to note, however, that even in complete darkness the eyes continually make saccadic movements,^39^ and it is therefore plausible that palisade ending development depends on eye movements per se, although not those elicited by visual stimuli.

### Eye Movements Drive Palisade Ending Development

To evaluate the impact of eye movements on palisade ending development, we injected BoNT-A into the retrobulbar space of the kittens at P0. BoNT-A is a poison that blocks the release of acetylcholine at the neuromuscular junction.^40^^,^^41^ The paralytic effect of this neurotoxin has been demonstrated in the skeletal muscles of rat^42^ and eye muscles of cat.^43^ Because BoNT-A weakens the contractile properties of an overacting muscle, it is often used to treat various forms of strabismus as an alternative to surgery.^44^

Through retrograde axonal transport, BoNT-A can also produce effects in motoneuron cell bodies.^45^ Specifically, BoNT-A injection into the lateral rectus muscle of cats reduces the synaptic coverage and firing pattern of abducens motoneurons.^25^^,^^45^ Because of these central actions, we cannot exclude the possibility that BoNT-A also caused some effects that directly or indirectly influenced the developmental program of the axon forming palisade endings. Developing and regenerating axons rely on neuronal activity,^46^ but it has also been demonstrated that only a single session of electrical stimulation is sufficient to enhance axonal regeneration,^47^^,^^48^ and this speaks more for an on/off switch in the growing and regeneration program of axons. This is why we consider that reduced eye motility caused by the neuroparalytic agent BoNT-A is likely to be responsible for the development features of palisade endings in the BoNT-A paradigm.

Only a single study^49^ has analyzed the effect of BoNT-A on palisade endings but experiments were done in adult cats, when palisade endings are already mature, whereas the present study was done in newborn kittens when palisade endings are developing. After BoNT-A treatment, palisade endings in adult cats exhibit changes at the fine structural level, including increase of structural proteins (neurofilament) in myelinated and unmyelinated axons, separation of the myelin sheath, and incomplete coverage of terminal varicosities with Schwann cells.^49^ It is important to note, however, that terminal varicosities of palisade endings are usually incompletely covered by Schwann cells,^8^^,^^9^^,^^13^ and this feature cannot be a consequence of the BoNT-A treatment. Thus, the neurotoxin-induced changes of adult palisade endings are probably less severe than claimed in the previous study.^49^ We observed a strong response in developing palisade endings after BoNT-A administration at P0. Specifically, palisade endings were absent in the superior rectus and lateral rectus muscles 45 and 95 days after birth and were only present in the inferior rectus and medial rectus muscles. This is in contrast to the control side where palisade endings were present in all rectus muscles. Structurally, palisade endings in the BoNT-A–treated inferior rectus and medial rectus muscles were rudimentary at the age of P45 and P95. Finally, the number of palisade endings was much lower on the injected than the control side. This suggests that, under the influence of BoNT-A, fewer axons extended into the tendon to form palisade endings. Altogether, these distinct qualitative and quantitative changes demonstrate that palisade ending maturation is severely hampered following BoNT-A treatment.

By visualization of GAP43, we tested whether the delay in palisade ending development is also visible at the molecular level. GAP43 is a neuronal growth protein^50^ and highly expressed in immature palisade endings but poorly expressed in mature palisade endings.^19^ This was confirmed in the present study because the GAP43 levels were higher in palisade endings of a P22 cat than in palisade endings of a P95 cat on both the control and the BoNT-A injected sides. By quantification of GAP43 in the P95 cat (after P0 BoNT-A injection), we observed that the GAP43 expression was weaker on the injected side than the control side. This result was surprising because we would have expected that GAP43, as indicator of the degree of maturation, was upregulated in BoNT-A–treated palisade endings, if they were developmentally delayed. Because the GAP43 levels at P95 in BoNT-A–treated palisade endings have been shown to be lower than in controls, and because palisade endings of the medial rectus muscle at P95 already show similar characteristics to the adults,^17^ altogether this would imply that no more axonal growth is expected in P95 palisade endings treated with BoNT-A. This finding is different from that of a previous study of developing chick EOM,^51^ where it has been observed that the structure and function of the muscles recover by 3 to 4 months after BoNT-A treatment. We did not observe signs of recovery because palisade endings were still rudimentary 95 days (3 months) after BoNT-A injection. It is therefore possible that BoNT-A–treated palisade endings do not reach adult-like levels or, alternatively, that the axonal recovery process in cat is longer than in chick.

There is evidence that palisade endings are formed by MIF motoneuron axons that establish several en grappe motor terminals alongside muscle fibers.^16^^,^^18^ We checked en grappe motor terminals associated with palisade endings after BoNT-A treatment on the control and injected sides. Interestingly, unlike palisade endings, en grappe motor terminals exhibited no structural alterations at the light microscopic level following BoNT-A treatment. Because palisade endings are peripheral expansions of MIF motoneuron axons,^16^^,^^18^ this would imply that the peripheral part of MIF axons forming palisade endings is more affected by BoNT-A than the central part forming en grappe motor terminals. Further studies are necessary to elucidate the effect of the neurotoxin on en grappe motor terminals.

The development of palisade endings follows a muscle-specific time course^19^ to reach a muscle-specific final density.^19^^,^^52^ That is, palisade endings are fully developed earlier and are more numerous in the medial rectus than in the other rectus muscles,^19^ which was confirmed in controls of the present study. Interestingly, this specific pattern of palisade ending development and distribution was retained following BoNT-A treatment because palisade endings were further developed and more numerous in the medial rectus muscle, even though they never reached adult-like quality and quantity. This observation suggests that palisade ending maturation and distribution basically follow an intrinsic program, but external perturbation (eye immobilization) can interfere with the time course of this program. Palisade endings develop in the first month of life,^19^ and the present study demonstrated that, during this period, palisade endings are extremely vulnerable to disturbances of the oculomotor activity. In other words, our findings provide evidence that during a critical postnatal time window eye movements have a crucial effect on palisade ending maturation.

### Palisade Endings and Strabismus

Infantile (congenital) strabismus is a misalignment of the eyes and disrupts the establishment of binocular vision. The pathogenesis of this disease is still unclear, but one possible cause is an unbalanced functional relationship between an antagonistic muscle pair due to an overactive or underactive muscle (for review, see Ref. 53). Typically, infantile strabismus develops in the first 6 months after birth.^54^ In humans, palisade endings are mature at the age of 2 years.^55^ Two studies^56^^,^^57^ have investigated palisade endings in subjects with infantile horizontal strabismus (eso- and exotropia). Variable changes ranging from mild to severe were detected in all palisade endings of the horizontal eye muscles (medial rectus and lateral rectus muscles),^56^^,^^57^ and various levels of altered palisade endings co-exist in the same muscle.^56^ These findings in infantile strabismus are comparable to the present findings, where we showed that BoNT-A–induced muscle paralysis in the first month of life altered the palisade ending phenotype. This underlines the notion that palisade endings are very sensitive to muscle perturbations in the early postnatal period, which is supported by findings in subjects with acquired strabismus, where palisade endings exhibit no significant structural changes.^57^ As opposed to infantile strabismus, acquired strabismus appears later in life at a time when palisade endings are already mature and probably, less vulnerable to oculomotor disorders.

Today, the functional significance of palisade endings is still elusive due to the fact that palisade endings combine both sensory^8^^,^^10^ and motor^13^^,^^16^^–^^18^^,^^52^ features. Irrespective of whether palisade endings are sensory or motor, there is supporting evidence that they play a role in convergence eye movements because the speed of development and density are higher in the medial rectus muscle than in the other rectus muscles.^7^^,^^19^ Moreover, many palisade endings in the medial rectus and some in the inferior rectus muscle of primates (human and monkey) express calretinin, and it is hypothesized that calretinin-positive palisade endings represent a specialized, probably more excitable type of palisade ending required for convergent eye movement.^15^^,^^52^ In the cat, a frontal-eyed species, palisade endings are immature at birth, and the present study has shown that eye movements in the early postnatal period are crucial for the development of palisade endings. Given that such an essential stimulus also drives the development of palisade ending in humans, this should be considered when designing therapies for oculomotor disorders.
